# Body density and diving gas volume of the northern bottlenose whale (*Hyperoodon ampullatus*)

**DOI:** 10.1242/jeb.137349

**Published:** 2016-08-15

**Authors:** Patrick Miller, Tomoko Narazaki, Saana Isojunno, Kagari Aoki, Sophie Smout, Katsufumi Sato

**Affiliations:** 1 Sea Mammal Research Unit, University of St Andrews, St Andrews, Fife KY16 9QQ, UK; 2 Atmosphere and Ocean Research Institute, University of Tokyo, 5-1-5 Kashiwanoha, Kashiwa, Chiba 277-8564, Japan

**Keywords:** Body condition, Lipid, Hydrodynamic performance, Drag, Buoyancy

## Abstract

Diving lung volume and tissue density, reflecting lipid store volume, are important physiological parameters that have only been estimated for a few breath-hold diving species. We fitted 12 northern bottlenose whales with data loggers that recorded depth, 3-axis acceleration and speed either with a fly-wheel or from change of depth corrected by pitch angle. We fitted measured values of the change in speed during 5 s descent and ascent glides to a hydrodynamic model of drag and buoyancy forces using a Bayesian estimation framework. The resulting estimate of diving gas volume was 27.4±4.2 (95% credible interval, CI) ml kg^−1^, closely matching the measured lung capacity of the species. Dive-by-dive variation in gas volume did not correlate with dive depth or duration. Estimated body densities of individuals ranged from 1028.4 to 1033.9 kg m^−3^ at the sea surface, indicating overall negative tissue buoyancy of this species in seawater. Body density estimates were highly precise with ±95% CI ranging from 0.1 to 0.4 kg m^−3^, which would equate to a precision of <0.5% of lipid content based upon extrapolation from the elephant seal. Six whales tagged near Jan Mayen (Norway, 71°N) had lower body density and were closer to neutral buoyancy than six whales tagged in the Gully (Nova Scotia, Canada, 44°N), a difference that was consistent with the amount of gliding observed during ascent versus descent phases in these animals. Implementation of this approach using longer-duration tags could be used to track longitudinal changes in body density and lipid store body condition of free-ranging cetaceans.

## Abstract (precis)

**Summary:** Body density and diving gas volume, two important but poorly understood physiological characteristics of beaked whales, are revealed through analysis of hydrodynamic performance during glides.

## INTRODUCTION

Body condition is expected to affect and be affected by numerous aspects of the behavioral ecology of animals, including anti-predator, foraging, migration and reproductive behavior (McNamara and Houston, 1990; Houston et al., 1993; Miller et al., 2012a; Crossin et al., 2014). Because of the influence of body condition on reproduction and survival, the capability to measure body condition of free-ranging cetaceans has the potential to be helpful in designing conservation efforts for large whales which are otherwise difficult to study (Hunt et al., 2013). Recent research has indicated that beaked whales are particularly sensitive to anthropogenic disturbance (e.g. from anthropogenic underwater noise: DeRuiter et al., 2013; Moretti et al., 2014; Miller et al., 2015). Measurement of body condition of beaked whales may be particularly helpful as a research tool to quantify the risk that disturbance (from noise or other sources) or habitat changes could lead to a reduced ability of individuals to grow, reproduce or survive as a result of effects on their energy budget (NRC, 2005; New et al., 2013).

Body condition can be quantified in numerous ways, but is typically defined as energy stored in the lipid fat reserves carried by an individual (Stevenson and Woods, 2006). Lipid store body condition (i.e. the quantity of lipid carried by an animal) reflects an integration of metabolic costs, foraging success and reproductive expenditure of the individual in their life cycle (Kleiber, 1961). For example, for seals that breed on land, researchers have been able to quantify the resources required to produce offspring and the consequences of variation in resources on future survival and reproductive success (Pomeroy et al., 1999; McMahon et al., 2000). ‘Capital-breeding’ seals, such as the British grey seal (*Halichoerus grypus*), fast while breeding, and female fat stores predict reproductive success (Hall et al., 2001). Measuring the body condition of wild cetaceans is inherently more challenging than for seals because live animals are not accessible on land. Work on blubber thickness in the Northern right whale (*Eubalaena glacialis*) indicates that blubber stores vary with reproductive status (Miller et al., 2011). Visual assessment of body condition appears to be effective for determining body condition of certain whale species (Bradford et al., 2012).

Net buoyancy (the difference between the mass of the body and the displaced water medium) of breath-hold divers affects their swimming behavior and energetics (Williams et al., 2000). The total density of the diver depends upon the density of relatively incompressible non-gas tissues (tissue density), and the volume and density of highly compressible gas stores carried within the body. The volume of gas carried within the body depends upon the diving lung volume, which is defined as the quantity of air carried in the lung of an animal at the start of a dive. Diving lung volume is an important parameter in respiratory physiology because it affects the available oxygen store for aerobic metabolism during dives (Ponganis, 2012) and predicted levels of nitrogen absorption (Hooker et al., 2012) during a dive. Because lipids are less dense than other non-gas body components, body density is strongly affected by lipid store body condition (Biuw et al., 2003; Fields et al., 2005; Moya-Laraño et al., 2008). For marine divers, variations in net animal buoyancy driven by body density and diving gas volume lead to differences in swimming and gliding patterns during dives (Sato et al., 2002, 2003; Miller et al., 2004; Nousek-McGregor et al., 2014). Thus, physiology and locomotion are linked because of the influence of gas and fat stores on the buoyancy force acting upon breath-hold divers.

The effect of lipid stores on the buoyancy of seals led to the development of a widely used method to longitudinally track the lipid store body condition of free-ranging elephant seals (*Mirounga leonina*) based upon the direction and rate of movement during passive drift dives (Biuw et al., 2003). The ‘drift rate’ is the speed at which the force generated by the net buoyancy of the animal (resulting from the difference of body density from the density of the surrounding water medium) is exactly offset by the force of drag resisting the movement. Sensitivity analyses indicated that the drag coefficient was a critical parameter for relating drift rates to body lipid stores in elephant seals (Biuw et al., 2003). Drift rates of elephant seals, as a reflection of lipid store body condition, have been useful to describe where in the marine environment elephant seals gain critical lipid resources (Biuw et al., 2007; Robinson et al., 2010), and how net buoyancy might affect their swimming behavior and energetics (Adachi et al., 2014; Jouma'a et al., 2016).

List of symbols and abbreviations

*a*: acceleration, change in speed (m s^−2^)
*A*: surface area (m^2^)
ARTS: aerial rocket transmitting system
*C*_d_: drag coefficient (unitless)
CI: credible interval
CTD: conductivity–temperature–depth
*d*: depth (m)
DIC: deviance information criterion
***g***: acceleration due to gravity (9.8 m s^−2^)
*m*: mass of the whale (kg)
*p*: pitch angle (rad)
ρ_air_: density of gas (kg m^−3^)
ρ_sw_: density of seawater (kg m^−3^)
ρ_tissue_: density of non-gas component of the whale body (kg m^−3^)
*r*: compressibility (proportion)
*v*: speed (m s^−1^)
*V*_air_: volume of air carried by the animal (ml)

Unfortunately, the drift dive method is not a generally applicable approach to estimate lipid stores of all marine mammals, as few species have been shown to perform such passive resting dives at sufficient depth to avoid overwhelming effects of gas-driven buoyancy (Lesage et al., 1999; Page et al., 2005). However, shorter gliding periods during descent and ascent phases of dives is a common behavior, with more gliding occurring in the movement direction aided by net buoyancy (Williams et al., 2000; Miller et al., 2004). Gliding periods can be identified unequivocally from animal-attached tags that sample accelerometers at more than twice the stroke frequency, which register animal thrusting movements (Sato et al., 2003). As no thrust is being produced by the animal during gliding periods, the speed and acceleration of the gliding body is a predictable outcome of identified external forces (drag, buoyancy) acting upon it. Well-established hydrodynamic equations predict how drag and buoyancy forces influence speed performance during glides, which has enabled quantification of body density of divers using analysis of short-duration glides (Miller et al., 2004) or longer-duration terminal velocity glides (Watanabe et al., 2006). Aoki et al. (2011; fig. 4 therein) found strong correspondence in body density of diving elephant seals estimated using vertical rates during drift dives, terminal velocity during prolonged glides, or change in speed during short glides.

Here, we used hydrodynamic analysis of short gliding periods during descent and ascent phases to quantify the diving lung volume and body density of deep-diving (Hooker and Baird, 1999) northern bottlenose whales, *Hyperoodon ampullatus* (Forster 1770), in two distinct north Atlantic habitats (Jan Mayen, Norway, and the Gully, Nova Scotia, Canada). Our goal was to provide the first estimates of these key physiological parameters for this species of beaked whale. Though the volume of an excised lung has been measured for *Hyperoodon* *ampullatus* (Scholander, 1940), no study has quantified the volume of gas carried to depth by any beaked whale. Tissue density of this species is a crucial determinant of its net weight in water, and variation across and within individuals is likely to reflect the volume of lipid stores carried by each animal, and to be influenced by various life-history and environmental parameters. We describe how precisely body density can be estimated, how it is influenced by compression at the depths experienced by beaked whales, how it relates to gliding patterns during descent and ascent phases of dives, and some patterns of variability across individuals.

## MATERIALS AND METHODS

Field studies were carried out in the Gully Marine Protected Area (hereafter, the Gully) off Eastern Canada from F/V *On A Mission* in July 2011 and 2013, and off Jan Mayen, Norway, in 2013 from the M/S *HU Sverdrup II* and in 2014 from the 29 m T/S *Prolific*. Conductivity–temperature–depth (CTD) casts were made in the Gully on 4 September 2013 at 43°49.166N, 58°52.164W. CTD casts were made off Jan Mayen on 24 June 2013 at 70°47.154′N, 6°0.473′W, and temperature-only casts were made off Jan Mayen in 2014 near each tag location. CTD and temperature cast data were converted to ambient water density following the standard international thermodynamic equation of state for seawater (Millero, 2010).

Whales were detected at sea either visually or by acoustic monitoring of their click vocalizations with a towed array. Animals were not pre-selected for tagging, but tagging was attempted on any animal except small calves that surfaced within tagging range. Fieldwork was conducted with research permits from Norwegian and Canadian animal research authorities, including permission to operate in protected waters in the Gully and near Jan Mayen. All research protocols were approved by the animal research ethics committee of the School of Biology at the University of St Andrews.

Animal-attached data loggers were either 3MPD3GT loggers (Little Leonardo, Co.) or sound and movement recording DTAGs (Table 1) attached using suction cups. The 3MPD3GT logger sampled depth, temperature, 3-axis magnetism and speed from a flywheel at 1 Hz sampling rate as well as a 3-axis ±3 ***g*** accelerometer at 32 Hz. The DTAG sampled pressure and a 3-axis ±2 ***g*** acceleration at 50 Hz, which was later downsampled to 5 Hz. Tags were attached to individuals using either a 5 m hand pole or an aerial rocket transmitting system (ARTS), which has a greater effective tagging range to 12–15 m. Tagging was conducted from the vessel *On A Mission* or *Prolific*, or from workboats deployed from *Sverdrup* and *On A Mission*. In 2014, bottlenose whales off Jan Mayen were tagged using the ARTS launching system directly from the deck of the *Prolific*. A desired sample of six whales from each of the two study locations was collected prior to completing these analyses, and no whale records were excluded from the analyses.

**Table 1.**
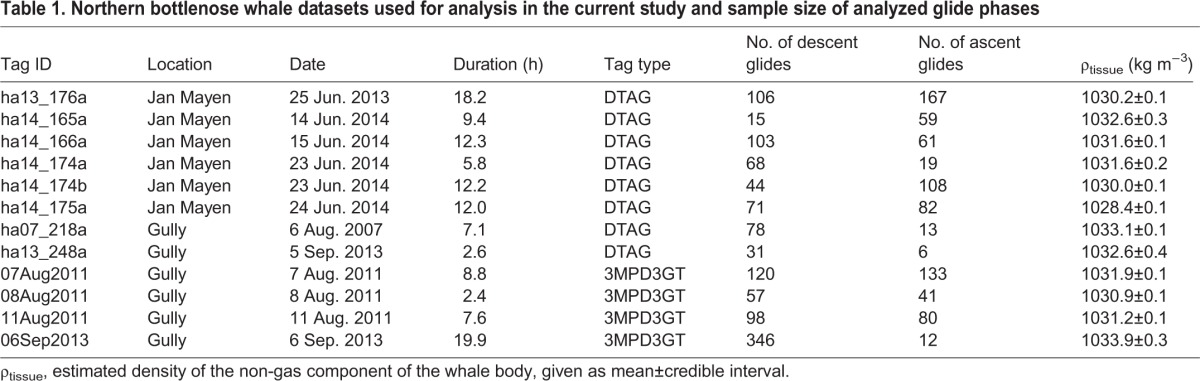
**Northern bottlenose whale datasets used for analysis in the current study and sample size of analyzed glide phases**

### Tag-data analysis

Pressure data recorded by the archival loggers were converted to absolute values of pressure using calibration values. Propeller rotations on the 3MPD3GT were converted to speed through the water using a calibration line for each tag deployment (Sato et al., 2003): a linear regression of rotation rate against swim speed that was calculated from mean vertical depth change divided by mean sine of the pitch at 5 s intervals when the mean absolute sine of the pitch was >0.9 for the deployments in 2011 and >0.8 for deployments in 2013. The *R*^2^ values from the linear regressions ranged from 0.72 to 0.84, and speed resolution was 0.019–0.025 m s^−1^. Tags were attached to whales at random orientations, so the tag-frame 3-axis acceleration data recorded by the loggers was converted to whale frame using established methods (Johnson and Tyack, 2003; Miller et al., 2004). Animal pitch from −90 to +90 deg was calculated as the arcsine of the dorsal–ventral axis of the animal-frame accelerometer. The DTAG lacks a speed sensor, so speed during glides was estimated using the rate of change of depth divided by the sine of pitch (Miller et al., 2004).

Dives were broken into descent, bottom and ascent phases following Miller et al. (2004). Gliding periods within each tag record were detected automatically and checked by hand. To detect glides automatically, the dorsal–ventral accelerometer data were high-pass filtered at 0.19–0.25 Hz to reduce gravitational components. Stroking was identified when oscillation on the dorsal–ventral axis of the accelerometer exceeded a threshold that was set for each deployment (0.1–0.5 m s^−2^).

Gliding data were extracted for 5 s duration segments. Glides longer than 5 s were broken into 5 s segments and every other 5 s segment within those longer glides was excluded from analysis to reduce autocorrelation. Acceleration during the glide (*a*) was measured by regressing speed versus time over the 5 s segments. The variance of the measurement was quantified as the root mean square sum of residuals from the fit of speed versus time (acceleration). Mean speed (*v*), depth (*d*) and pitch angle (*p*) were calculated for each 5 s glide segment. Seawater density (ρ_sw_) for each glide segment was calculated from the CTD cast that was closest in time to each tag record, but was updated with seawater temperature measurements from temperature-only casts or recordings on the 3MPD3GT logger deployments on whales. To avoid the influence of animal maneuvering on speed performance during a glide, only stable glides (circular variance of roll <0.1) were included. Only glides during the descent and ascent phase that were at 30 deg pitch angle or steeper were included to enable robust estimates of speed for DTAG records.

### Model for hydrodynamic performance

Eqn 1 relates acceleration (*a*) during glides to the forces of drag and net buoyancy of tissue and gases carried by each whale (Miller et al., 2004). Note that effects of lift and associated induced drag (if present) are ignored in this equation (see Aoki et al., 2011):
(1)
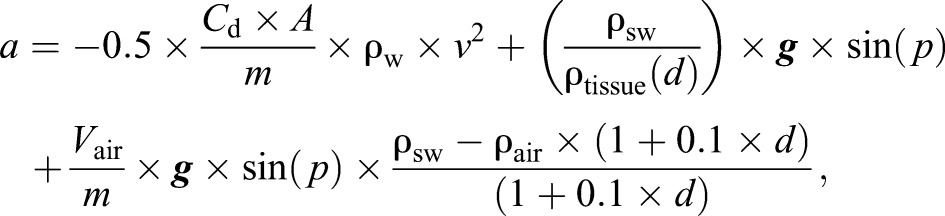


where:

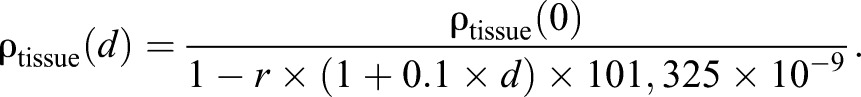


Here, *C*_d_ is the drag coefficient, *A* is the relevant surface area (m^2^), *m* is the mass of the whale (kg), ρ_sw_ is the density of the surrounding seawater (kg m^−3^), ρ_tissue_ is the density of the non-gas component of the whale body (kg m^−3^), ***g*** is acceleration due to gravity (9.8 m s^−2^), *p* is animal pitch (rad) with negative values indicating a downward orientation, *V*_air_ is the volume of air carried from the sea surface (ml), ρ_air_ is the density of air (kg m^−3^), *d* is glide depth (m) and *r* is compressibility of the animal tissue or the fractional change in volume per unit increase in pressure. The value 101,325 converts pressure in atmospheres to pressure in Pa, so that the units of body tissue compressibility are proportion×10^−9^ per Pa. The equivalent compressibility value for 0°C water of salinity 35 ppm is 0.447×10^−9^ Pa^−1^.

Thus, the model consists of three terms that represent external forces acting on the gliding body: drag, density of tissue relative to the surrounding seawater, and air volume. The first term quantifies the effect of drag on the speed of the whale during a glide, which always acts against the direction of movement of the body. The effect of drag is primarily a function of speed and unknown terms (*C*_d_*A*)/*m*, which are treated together in this paper with units of m^2^ kg^−1^.

The second and third terms relate to the weight of the body (net buoyancy) in water, and can act either with or against the direction of movement of the body. Total body density is the total mass of both tissue and gas components divided by their volumes. Because net buoyancy operates vertically, the component along the movement axis of the whale is obtained by multiplication by sine of pitch (*p*).

The second term quantifies the influence of unknown non-gas tissue density (ρ_tissue_) on speed during a glide. The value for this term is expected to vary across individuals depending upon their lipid store body condition. While the temperature of the whale body is not expected to follow ambient conditions, the whale will experience local pressure effects, and though the tissue compartment of the body should be relatively incompressible compared with gas compartments, some compression is expected at the extreme pressures experienced by these divers. Previous studies have assumed this compressibility factor to be similar to that of seawater (Miller et al., 2004). However, our model in the present study explicitly accounts for tissue compressibility by multiplying the tissue density by the whale tissue compressibility factor (*r*).

The third term quantifies the influence of the unknown volume of gas per unit mass carried in the dive (*V*_air_/*m*) on the net buoyancy of the diver. Gas compartments of cetaceans are largely unprotected from ambient pressure conditions. Thus, the volume and density of gas carried by the animal were modelled to change with hydrostatic pressure following Boyle's law.

### Bayesian estimation procedure

Data extracted for each 5 s glide segment were used to estimate unknown parameters in the hydrodynamic performance model (Eqn 1) using Bayesian Gibbs sampling with freely available software JAGS within R (coda, R package v0.17-1 2015, https://cran.r-project.org/web/packages/coda/index.html) and R2jags (R package v0.5-7 2012, https://cran.r-project.org/web/packages/R2jags/index.html). Bayesian estimation assumes that parameters are random variables with a ‘prior’ distribution (our *a priori* expectation of what the parameter distribution should be), as opposed to traditional frequentist estimation that assumes parameters are unknown and fixed. The Gibbs sampling algorithm seeks to estimate the posterior distribution, which is the best estimate of the true parameter distribution after the prior expectation of this distribution has been updated with data (Lunn et al., 2012).

We chose the Bayesian over frequentist methods to allow for more flexibility in the statistical model development. A key innovation of the statistical procedure was the inclusion of nested (hierarchical) parameters to contrast within- and across-individual variability, which was more straightforward to implement using the MCMC algorithm. The Bayesian estimation framework also allowed us to implement models with informative priors so that we could include information from other studies and *a priori* reasoning to support the model-fitting process. We compared the results with fits using uninformative priors to determine the sensitivity of the process to assumptions about the priors. Finally, instead of using traditional regression analysis where the sum of squared errors is minimized, we were able to implement observation error for the measured acceleration values. This weighted the high-quality over low-quality acceleration data in the estimation.

There were four unknown terms in the equation, each of which was set a specific prior range. Compressibility (*r*) was set a uniform (non-informative) prior from 0.3×10^−9^ to 0.7×10^−9^ Pa^−1^. Body tissue density (ρ_tissue_) was set a uniform prior from 800 to 1200 kg m^−3^. Diving gas volume was set a uniform prior from 5 to 50 ml kg^−1^. For the combined drag coefficient term [(*C*_d_*A*)/*m*] (Biuw et al., 2003), several sources of data were used to set an informative prior. Drag coefficient was estimated to be roughly 0.0030 based upon previous research on similar-sized large cetaceans (killer whale, *Orcinus orca*: 0.0029 – Fish, 1998; fin whale, *Balaenoptera physalus*: 0.0026 – Bose and Lien, 1989; sperm whale, *Physeter microcephalus*: 0.0031 – Miller et al., 2004). Based upon body length ranges from 5.8 to 9.8 m, surface area (mean 23.0 m^2^, range 12–36 m^2^) and mass (mean 6816 kg, range 3027–12,739 kg) were estimated using the equation derived for sperm whales (Miller et al., 2004). This led to an expected value for the northern bottlenose whale for the combined [(*C*_d_*A*)/*m*] term of 10×10^−6^* *m^2^* *kg^−1^, with a range from 8×10^−6^* *m^2^* *kg^−1^ for large animals to 12×10^−6^* *m^2^* *kg^−1^ for small animals. We captured uncertainty in this mean value by specifying the prior to be a normal distribution with a mean of 10.0×10^−6^* *m^2^* *kg^−1^ and standard deviation of 2.0×10^−6^* *m^2^* *kg^−1^. The distribution for (*C*_d_*A*)/*m* was truncated at 5.0×10^−6^ and 20.0×10^−6^ m^2^ kg^−1^ to limit the range of values explored by the Bayesian sampling algorithm.

A set of models were evaluated in order to explore variability in body density, the drag term and diving lung volume. For each of these quantities, we considered models in which the quantity remained constant across the tags and dives (global estimates), and models with individual-specific estimates for each tag record. For diving lung volume, we also considered a dive-specific as opposed to individual-specific model. We also fitted hierarchical models with a global parameter included. In these hierarchical models, each individual or dive-by-dive estimate was considered to be a sample from a global or ‘population’ distribution with an estimated global mean, and an estimated variance across the dives and individuals. Thus, this model structure assumed a central tendency (a shared mean and variance) to the distribution of dive-specific and individual-specific values. A detailed structure of the hierarchical model is given in the JAGS script (see Script S1).

With global parameters, the estimation routine was able to borrow strength across the tag records and different dives to estimate the individual and dive-by-dive parameters in a hierarchical model structure. The global distribution also has a meaningful interpretation as the population distribution for that parameter. For body density, for example, the global distribution is the expected distribution of body density in the population of whales from which the tagged whales were sampled.

All models were sampled in three independent chains, with 24,000 iterations each. The first 12,000 samples were discarded for burn-in, and the remaining posterior samples were downsampled by a factor of 36. Convergence was assessed for each parameter, using trace history and Brooks–Gelman–Rubin diagnostic plots (Brooks and Gelman, 1998). Model selection was based upon the deviance information criterion (DIC), with a lower value indicating a better model fit relative to model complexity.

## RESULTS

We recorded fine-scale movements of 12 northern bottlenose whales (Table 1). In the Gully, four 3MPD3GT loggers and two DTAGs were deployed, while only DTAGs were deployed in Jan Mayen. In the Gully, all tags were attached using 5 m hand poles while tags in Jan Mayen were attached using a remote tag launching system (ARTS; Kvadsheim et al., 2014), which enabled tag attachment at greater distances than is possible using a hand pole.

Seawater temperature and corresponding water density measured by CTD differed strongly between Jan Mayen and the Gully. Jan Mayen had an almost constant temperature profile near 3°C, with a sea-surface temperature of 5–6°C. In contrast, the Gully had a much warmer sea-surface temperature of 17°C, with a stratified temperature profile down to 400 m depth when it became constant near 5°C.

**Fig. 1.**
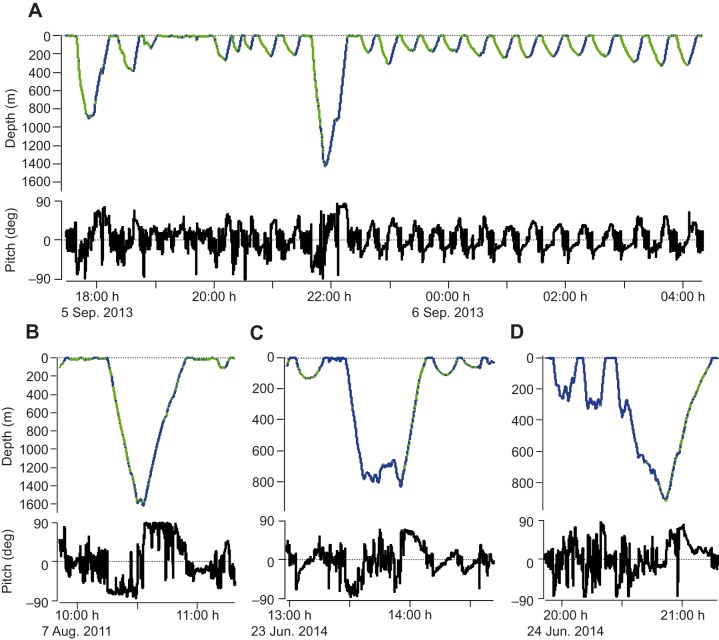
**Example** **data records for diving profile and pitch.** Top: dive profile with gliding periods indicated in green and stroking periods in blue; bottom: pitch. Examples are taken from whale subjects 06Sep2013 (A), 07Aug2011(B), ha14_174b (C) and ha14_175a (D).

Gliding periods were successfully identified in all tag records (Fig. 1). While all whales spent time gliding, there was substantial variation in the proportion of time spent gliding during descent versus ascent across the different tag records.

Bayesian model estimates (means of the posterior distributions) were compared across the different models, which differed in how body density, diving air volume and drag coefficient terms were allowed to vary between individuals and dives (Table 2). The worst-fitting (the highest DIC) model contained only global values for these three terms, indicating that there was substantial variation in these quantities both across and within individuals. The best model structure with the lowest DIC evaluated global plus individual variation in body density and drag terms, as well as global plus dive-by-dive variability in diving lung volume. The best-fitting model had a DIC value of 32,376 (Table 2), while the next-best model had a DIC value of 33,748, a difference of 1372 units. Importantly, tissue density estimates varied very little across model structures, with estimated tissue density varying by <3 kg m^−3^ across the evaluated set of models (Table 2).

**Table 2.**
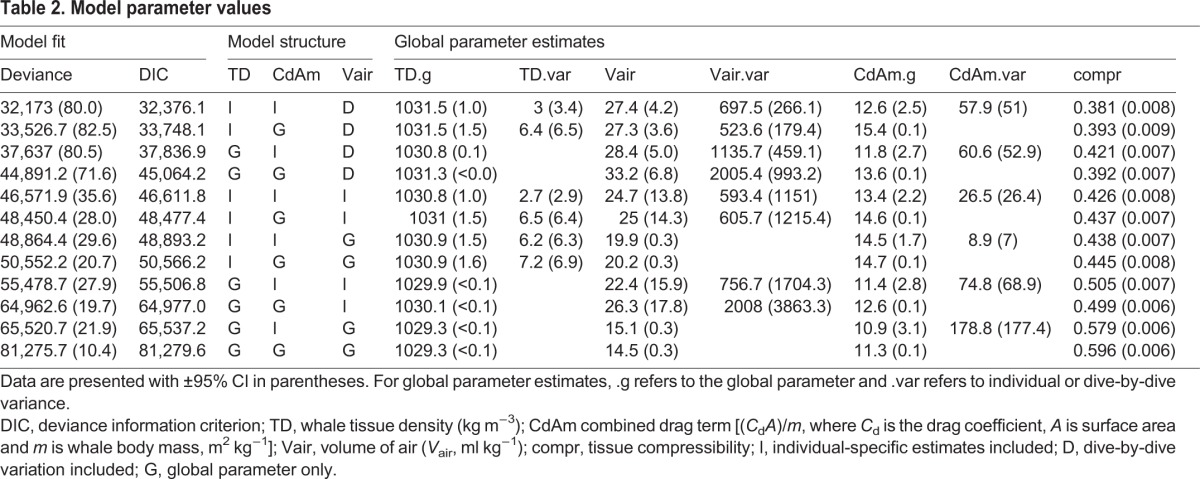
**Model parameter values**

**Fig. 2.**
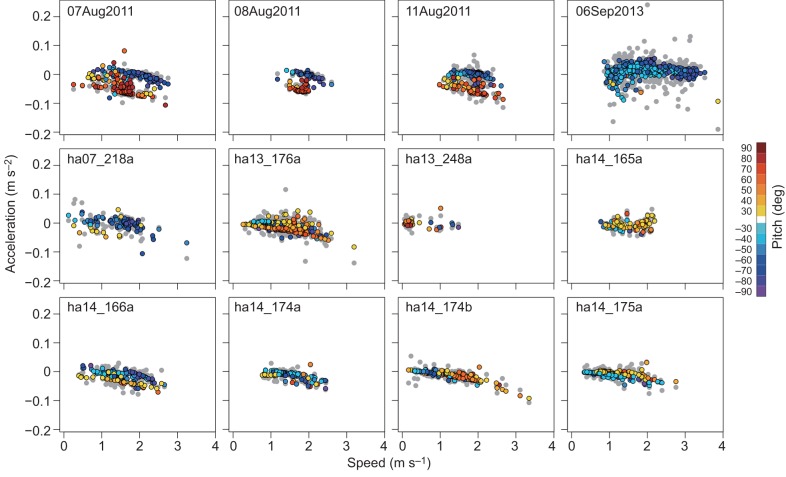
**Acceleration versus speed for each glide.** Observed acceleration is shown as gray circles. Posterior mean modelled acceleration is shown as colored circles; the color of each data point indicates the pitch angle recorded during the glide segment.

**Fig. 3.**
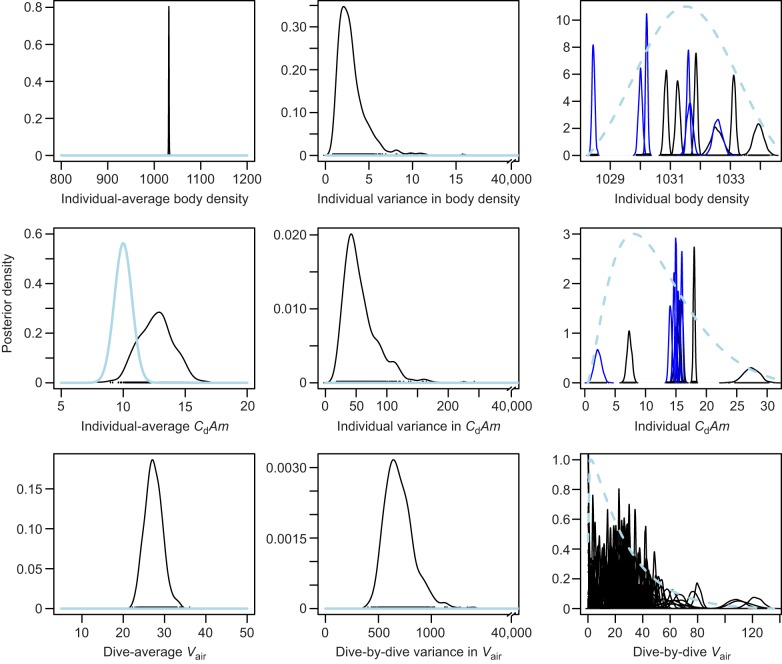
**Prior and posterior distributions from the best model with the lowest deviance information criterion (DIC).** The left and middle panels show the global or ‘population’ parameters (shared across all dives and individuals) and the right panel shows the individual and dive-specific parameters. Solid blue and black lines show the prior and posterior distributions, respectively. Note the *x*-axis break in the middle panel, which indicates the wide prior range for the between-group variances. Dashed blue lines (right panel) indicate the estimated global distributions, derived from the posterior mean value for the global average (left panel, e.g. individual-average body density) and the global variance (middle panel, e.g. inter-individual variance in body density). This distribution can be interpreted as the population distribution for that parameter.

We derived conclusions from the lowest-DIC model. This model provided a strong overall fit to the data, with a good match between observed and modeled (posterior mean) acceleration during glides (Fig. 2). Negative acceleration increased with glide speed, as predicted by the drag equation. Bayesian priors and posteriors are presented in Fig. 3.

Tissue compressibility was estimated as a single global parameter, with a posterior 95% credible interval (CI) of 0.37–0.39 (mean of 0.38), indicating somewhat lower compressibility than freshwater at 30°C. The best-fitting model had a DIC value of 32,376 (Table 2). Excluding the compressibility term from that model increased the DIC by 7181 units. This demonstrates that inclusion of the tissue compressibility term significantly improved the model fit to the measured data.

The posterior mean of the global drag term [(*C*_d_*A*)/*m*] was 12.6×10^−6^ m^2^ kg^−1^, overlapping, but slightly greater than the specified normal prior mean (Fig. 3). Most individual posterior means were near 15×10^−6^ m^2^ kg^−1^ and ranged overall from 2×10^−6^ to 27×10^−6^ m^2^ kg^−1^.

**Fig. 4.**
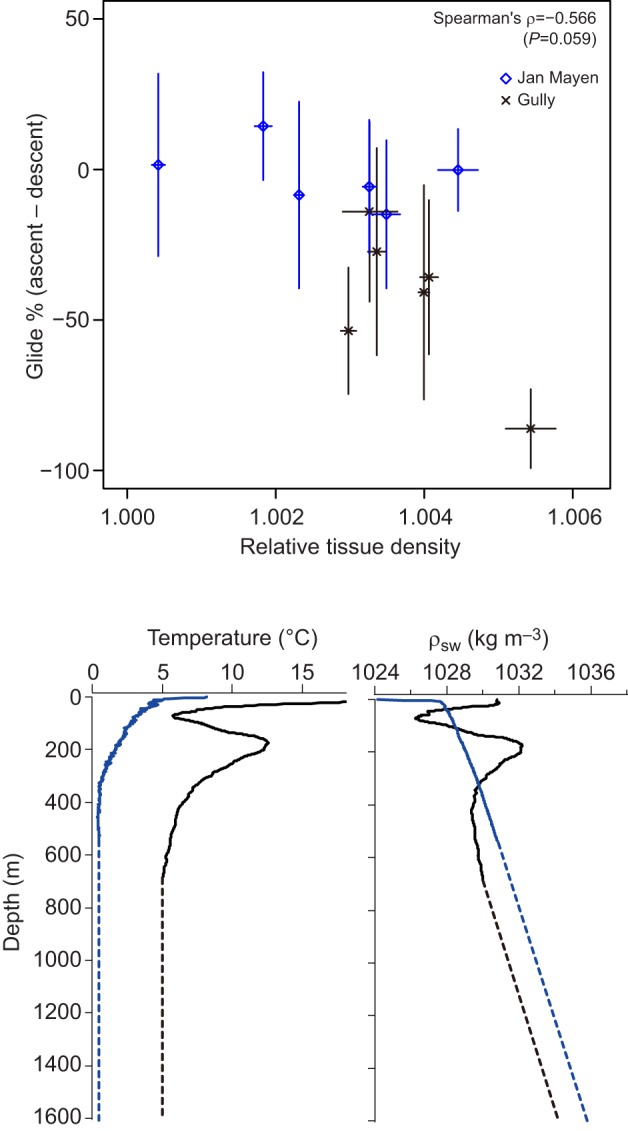
**Gliding patterns of whales versus tissue body density.** Top: difference in percentage of time spent gliding in ascent versus descent phases at depths >100 m as a function of tissue density of tagged northern bottlenose whales relative to ambient seawater density. Greater relative body density values indicate greater negative buoyancy of the whales, while values close to 1.0 indicate near-neutral buoyancy. Error bars on the *x*-axis indicate the 95% credible interval of body density; those on the *y*-axis indicate the standard deviation of gliding percentages across dives, with negative values indicating more gliding during the descent phase, and values near zero indicating equal proportions of gliding during descent and ascent phases. Note the strong expected relationship that denser animals glide relatively less during the ascent phase (more during descent), which is the transit direction aided by buoyancy. Bottom: temperature (left) and seawater density (ρ_sw_, right) profiles. Blue and black lines represent Jan Mayen (24 June 2014) and the Gully (4 September 2013) profiles, respectively. Conductivity–temperature–depth (CTD) profiles were measured up to around 600 m for both study sites. Salinity and temperature were assumed to remain constant at greater depths (dashed line).

The global mean tissue density was estimated at 1031.5±1.0 (95% CI) kg m^−3^. Individual posterior mean values for tissue density ranged from 1028.4 to 1033.9 kg m^−3^, with ±95% CI of 0.1–0.4 kg m^−3^ (Table 1). The proportion of time gliding during ascent versus descent phases correlated with tissue density, reflecting stronger negative net buoyancy during glides (Fig. 4, see also examples in Fig. 1).

Whales tagged in Jan Mayen had an overall tendency to be less dense than whales tagged in the Gully. Though several animals from both locations had values in the range 1030.5–1032.6 kg m^−3^, only whales from Jan Mayen had lower values while only whales from the Gully had greater values than that range. Mean values from the two locations differed by 1.5 kg m^−3^. This small but consistent difference in body density was clearly reflected in gliding patterns at depths >100 m, with animals in the Gully gliding substantially more during descent phases and Jan Mayen animals gliding more equally in descent and ascent phases (Fig. 4).

The mean global diving gas volume was estimated at 27.4±4.2 (95% CI) ml kg^−1^. The lowest DIC model also included dive-by-dive variation in the diving lung volume, with posterior mean estimates ranging from 0.1 to 121.9 ml kg^−1^ across the dives of all 12 whales. Individual averages of the dive-by-dive estimates ranged between 0.9 and 40.0 ml kg^−1^. However, there was no apparent relationship between estimated diving gas volume and dive duration or dive depth.

## DISCUSSION

In this study, we were able to effectively decompose the drag and buoyancy forces that operate on the beaked whale *H. ampullatus* body during gliding phases, enabling estimation of the species-typical volume of gas carried from the surface and each animal's tissue body density. Estimation of parameters in our hydrodynamic model (Eqn 1) led to a good match between measured and modelled acceleration values during glides (Fig. 2). The proportion of time spent gliding in the descent and ascent phases correlated as expected with body density (Fig. 4, see Fig. 1 for examples). Because denser animals are expected to glide more during descent than ascent, this provided a validation of our body density estimates external to the modelling approach.

The approach used in this analysis built upon methods used in previous studies dealing with sperm whales and elephant seals (Miller et al., 2004; Aoki et al., 2011). One improvement in the hydrodynamic model used here is that it includes an explicit term for tissue compressibility, which can be estimated from the data, rather than assuming that tissue compressibility is equal to compressibility of the surrounding seawater (e.g. Miller et al., 2004).

The value for compressibility of the whale was estimated as 0.38×10^−9^ Pa^−1^, somewhat less than the compressibility of 0°C seawater of 35 ppm salinity, and its inclusion was supported by model selection. The effect of this compression is quite minor in absolute terms, as the body is predicted to compress by only 0.3% at 1000 m depth. Thus, a whale with tissue density of 1031.0 kg m^−3^ at the sea surface would be predicted to have a body density of 1034.5 kg m^−3^ at 1000 m depth. Though the absolute value of the influence of compressibility on tissue body density is small, the effect of this compression on net buoyancy operating on the diver’s body is amplified as the density of seawater is very close to the tissue density of the whale. Therefore, inclusion of a tissue compressibility factor is important to effectively estimate body tissue density of divers that experience large changes in hydrostatic pressure when diving [Miller et al., 2004; K. Aoki, Diving behavior of sperm whales, PhD thesis (in Japanese), The University of Tokyo, 2008)].

Use of Bayesian estimation procedures made it possible to apply biologically informed prior information on important terms in the hydrodynamic model (Eqn 1). We used broad priors for unknown terms of interest – diving gas volume and body density – but specified a more narrow informative prior for the drag coefficient terms based upon available estimates for drag coefficient (*C*_d_) and mass (*m*) and surface area (*A*) of the tagged whales. We undertook this approach because of the quality of information available on the drag coefficient of similar-sized animals (Fish, 1998; Bose and Lien, 1989; Miller et al., 2004), and the range of body sizes of northern bottlenose whales.

We fitted a number of different models (Table 2), in order to evaluate the scale at which the unknown drag, gas and body density parameters vary across individuals, and for gas, also across dives. As it is driven by gross anatomical characteristics, tissue compressibility was assumed to be constant across whales and dives. The best model structure with the lowest DIC included global plus individual variation in body density and drag terms, as well as global plus dive-by-dive variability in diving lung volume. That result matches our expectation that body density and the drag term (*C*_d_*A*)/*m* may vary across individuals (Table 1). In contrast, diving gas volume could vary across individuals if there were sufficient variation in lung anatomy, or within tag records depending upon dive-by-dive behavioral decisions on inhalation. A substantially lower DIC score was obtained when diving gas volume was allowed to vary across dives (Table 2), indicating that dive-by-dive behavioural decisions on inhalation lead to greater variation than consistent differences across individual animals.

### Diving lung volume estimates

The global (dive-average) estimate for diving gas volume of *H. ampullatus* was 27.4±4.2 (95% CI) ml kg^−1^. Because the volume of gas changes most rapidly over shallow depths (Eqn 1), glides conducted near the surface provide the most information to estimate diving gas volume. Most shallow glides are conducted near the end of the ascent phase as expanding gases yield additional positive buoyancy (Fig. 1; see Sato et al., 2002; Miller et al., 2004). Thus, any release of gas during dives could cause our estimated diving gas volume to be lower than the true value taken from the surface. In our data, all of the acoustic DTAG deployments were carefully checked for acoustic evidence of bubble release (e.g. Hooker et al., 2005), and none was found. Thus, we conclude that our estimate reflects the quantity of the most compressible portion of gas taken down by the whale at the start of its dive.

Assuming the diving gas volume contracts within a pressure-compliant lung (Bostrom et al., 2008), 27.4±4.2 (95% CI) ml kg^−1^ represents the first estimate of the diving lung volume of a beaked whale. This value is similar to the total lung capacity of *H. ampullatus* measured from excised lungs, found to be 30–40 l of gas capacity for a 1400 kg whale or 21–29 ml kg^−1^ (Scholander, 1940). The value for northern bottlenose whales estimated here is roughly the same as the 26.4±3.9 ml kg^−1^ mass-specific diving gas volume previously estimated for the sperm whale (Miller et al., 2004).

A small mass-specific diving lung volume of *H. ampullatus*, compared with that of delphinids (Piscitelli et al., 2013), may be selected as a smaller diving lung volume is predicted to increase the level of pulmonary shunt and thereby reduce nitrogen uptake during dives of beaked whales (Hooker et al., 2009). The cost of such a small diving lung volume in terms of oxygen store is minor as most of their oxygen is carried in blood hemoglobin and muscle myoglobin stores (Ponganis, 2011). The lower limit of the quantity of gas carried to depth most likely reflects the requirement of these echolocating beaked whales to use gas to produce biosonar clicks during deep-dive foraging (Tyack et al., 2006). Based upon the results of this study, a 5000 kg animal is typically estimated to carry 137 l of air at the sea surface. Following Boyle's law, that volume of gas would be reduced to ∼1% of the surface value at a foraging dive depth of 1000 m (Fig. 1) leaving little more than 1 liter of gas for pneumatic echolocation click production at such foraging depths. There was no relationship between the estimated diving lung volume and dive depth (or duration), which is different from previous results obtained from penguins (Sato et al., 2002, 2011) and California sea lions (McDonald and Ponganis, 2012). Although no systematic variation with dive depth or duration was apparent in diving gas volume in this study, more detailed analyses of dive-by-dive variation in diving lung volume could reveal as-yet undetected strategies of gas utilization in beaked whales.

### Tissue density

An individual-average value of 1031.5±1.0 (95% CI) kg m^−3^ was estimated for the tissue density of *H. ampullatus* at a pressure of 1 atm experienced at the sea surface, indicating that non-gas body tissues are typically denser than seawater. Model selection supported across-individual variation in body density (Fig. 2). Body density of individuals estimated using the minimum DIC model ranged from 1028.4–1033.9 kg m^−3^ and was highly precise with ±95% CI of the posterior estimates ranging from 0.1 to 0.4 kg m^−3^, which would equate to a precision of <0.5% of lipid content based upon extrapolation from the elephant seal (Aoki et al., 2011). It is worth noting that values for body density were not very sensitive to model selection (Table 2), with values varying by <1 kg m^−3^ for most individuals. The sensitivity of body density estimates to model selection was lowest for tag records in which we recorded a large number of glides in both descent and ascent phases (Table 1). Unlike other beaked whales (Tyack et al., 2006), the northern bottlenose whales in this study did not ascend at shallow pitch angles (Fig. 1), so we were able to measure gliding performance during both ascent and descent glides for most individuals.

Our results identified a tendency for *H. ampullatus* tagged off Jan Mayen to have a lower body density than whales tagged in the Gully (Fig. 3). Data from CTD casts indicated that seawater densities in the warmer waters of the Gully were lower than those in Jan Mayen, with the result that denser *H. ampullatus* tagged in the Gully had greater net negative buoyancy than *H. ampullatus* tagged off Jan Mayen whose bodies were closer to neutral buoyancy (Fig. 4). Ascent versus descent gliding patterns were very different between the two locations, with most animals tagged near Jan Mayen gliding roughly equivalent proportions of time during descent and ascent (Fig. 4, top). In contrast, whales tagged in the Gully glided much more during descent phases of dives (Fig. 4, top).

The geographic difference in body density suggests that northern bottlenose whales tagged in the Gully had somewhat smaller lipid stores than whales tagged near Jan Mayen. It is not clear whether those differences might indicate a better feeding habitat off Jan Mayen, seasonal differences reflecting the different months that tags were attached, or simply different adaptive homeostasis for different levels of lipid store (McEwen and Wingfield, 2003). Greater fat stores for insulation may be adaptive for whales in the colder waters off Jan Mayen. Another explanation could be that animals off Jan Mayan increase energy stores prior to a southward migration. A bottlenose whale tagged with a SPLASH-10 satellite tag travelled from Jan Mayen to the Azores between 22 June and 4 August 2015 (R. Hansen and P.M., unpublished data). In contrast, northern bottlenose whales in the Gully are thought to be resident with less wide ranging patterns (Whitehead et al., 1997). More data are needed to identify factors that might drive these geographic and individual differences in lipid store. However, both theoretical (Miller et al., 2012b) and empirical (Sato et al., 2013; Adachi et al., 2014; Jouma'a et al., 2016) studies indicate that deviations of body density away from neutral buoyancy may entail greater swimming costs.

### Methods considerations

Our hydrodynamic model improves upon previous versions (Miller et al., 2004; Aoki et al., 2011) by enabling estimation of tissue compressibility. Linear effects of hydrostatic pressure on body density at depth were captured by the estimated tissue compressibility in the model. The effect of residual air on overall buoyancy is reduced at greater depths because air volume decreases with increasing depth following Boyle's law. For exhale divers, the effect is thought to become negligible at depths greater than 100 m (Biuw et al., 2003; see fig. 7 of Miller et al., 2004; Aoki et al., 2011). Therefore, glides conducted near the surface provide the most information for estimating diving gas volume. An estimate of residual air would be less reliable in cases with few shallow glides in data recorded for that specific dive. Our model assumed Boyle's law for the compression and re-expansion of gas in the body. It is possible that dissolved or small quantities of gases stored in certain tissues (e.g. within bone, muscle or brain) are relatively protected from ambient pressure and therefore compress and/or expand less than expected by Boyle's law alone.

The value of the drag term in the hydrodynamic equation (Eqn 1) varies with speed-squared, so the value of the combined drag parameter (*C*_d_*A*)/*m* can be confidently estimated from the data only if a wide coverage of gliding speeds is recorded in the data. This was the case in our data, but care should be taken when applying this method to other datasets to carefully assess the degree of data coverage. Using auxiliary published data to narrow the prior range of the combined drag parameter can improve the precision of estimates of body density in cases where the data coverage of gliding speed and/or pitch is limited. The effect of buoyancy depends on an animal's pitch (Eqn 1), and the relationship between acceleration and swim speed during glides varies with pitch (see fig. 2 of Aoki et al., 2011, for details). Therefore, estimation of body density is more robust if a wide coverage of gliding pitch is recorded. As in previous studies (Aoki et al., 2011), we neglected the possible addition of induced drag due to lift generation when animals glide at shallower pitch angles. Consideration of the possible influence of induced drag due to lift generation would be particularly important for datasets dominated by glides at shallow pitch angles.

While body density correlates strongly with total body lipid store content in mammals (Fields et al., 2005), conversion of a specific body density to an accurate value for lipid store content is only possible if the density of non-lipid tissues is known (Biuw et al., 2003). Density measurements of body components of cetaceans would be useful to more effectively convert total body density to lipid store values. Nonetheless, body density within a given species should provide a relative index of lipid store body condition across individuals (and potentially changes over longer time periods within individuals). Longitudinal tracking of changes in lipid stores using tags has been very effective in the study of resource acquisition and diving energetics of elephant seals (Biuw et al., 2007; Robinson et al., 2010; Adachi et al., 2014; Jouma'a et al., 2016). On-board implementation of the algorithm described in this work in a longer-duration telemetry tag (e.g. Schorr et al., 2014) could enable longitudinal tracking of body condition of individual beaked whales. Such studies could effectively aid conservation efforts by helping to identify effective foraging areas and quantifying the energy store impact of anthropogenic disturbance on the body condition of individual cetaceans.

One disadvantage of using body density to estimate body condition is the need to tag the subject whale, which will typically lead to a reduced sample size relative to the easier to implement visual (Bradford et al., 2012) or photogrammetry (Miller et al., 2012a; Durban et al., 2015) methods. However, the advantage of using body density to estimate lipid store body condition is that the method provides a quantitative, replicable indicator of the entire lipid store carried by an animal. Because of the presence of non-lipid materials within blubber, its thickness (Miller et al., 2011) may not vary linearly with lipid content, particularly when lipid stores are low. Measurement of body width versus length using overhead photogrammetry images has been shown to relate to nutritive body condition in some species (Miller et al., 2012a), but it is not clear whether such shape patterns are replicable for other more cryptic species. While ideal methods to estimate body condition of cetaceans may vary by species, the tag-based approach described here might be particularly useful for cryptic animals like beaked whales that are difficult to observe at sea.
